# The Gibbs Paradox: Lessons from Thermodynamics

**DOI:** 10.3390/e20050328

**Published:** 2018-04-30

**Authors:** Janneke van Lith

**Affiliations:** Department of Philosophy and Religious Studies, Utrecht University, Janskerkhof 13, 3512 BL Utrecht, The Netherlands; j.h.vanlith@uu.nl

**Keywords:** Gibbs paradox, thermodynamics, extensivity, foundations of statistical mechanics, indistinghuishability, entropy of mixing

## Abstract

The Gibbs paradox in statistical mechanics is often taken to indicate that already in the classical domain particles should be treated as fundamentally indistinguishable. This paper shows, on the contrary, how one can recover the thermodynamical account of the entropy of mixing, while treating states that only differ by permutations of similar particles as distinct. By reference to the orthodox theory of thermodynamics, it is argued that entropy differences are only meaningful if they are related to reversible processes connecting the initial and final state. For mixing processes, this means that processes should be considered in which particle number is allowed to vary. Within the context of statistical mechanics, the Gibbsian grandcanonical ensemble is a suitable device for describing such processes. It is shown how the grandcanonical entropy relates in the appropriate way to changes of other thermodynamical quantities in reversible processes, and how the thermodynamical account of the entropy of mixing is recovered even when treating the particles as distinguishable.

## 1. Introduction

There are in fact two distinct paradoxes that go under the heading of the Gibbs paradox. The original one was formulated by Josiah Willard Gibbs in 1875 [1]. It addresses the mixing of two quantities of ideal gas, and the entropy change that occurs as a result of the mixing process. The paradox arises from the difference between two scenarios: one in which two quantities of the same gas are mixed, and one in which the two gases being mixed are different kinds. First, of course, in the case of mixing two equal gases, thermodynamics tells us that there is no change of entropy. In this case, we could restore the initial thermodynamical state of the mixing process simply by putting the original partitioning back in place, at no entropy cost. It immediately follows that no change in entropy occurs when two quantities of the same kind of gas are mixed. Secondly, in contrast to this, when the two gases are of different kind, the final state of the mixing process is different from the initial state not only in a microscopic but also in a macroscopic description, and now an entropy increase does occur. It is well known that this entropy increase is equal to $2Nk_{B}\ln2$, a number which interestingly depends on the amounts of gases mixed, but not on their nature.

The paradoxical point, as noted by Gibbs, is that this leads to a discontinuity in the entropy of mixing, in an imagined sequence of mixing processes in which gases are mixed that are made more and more similar. Gibbs writes:

“Now we may without violence to the general laws of gases which are embodied in our equations suppose other gases to exist than such as actually do exist, and there does not appear to be any limit to the resemblance which there might be between two such kinds of gas. However, the increase of entropy due to the mixing of given volumes of the gases at a given temperature and pressure would be independent of the degree of similarity between them.”

[1] (p. 167).

Thus, in the imagined limit in which the gases become equal, the entropy of mixing drops to zero discontinuously. This is the original Gibbs paradox.

Both in textbooks on statistical physics and in the more recent literature on the subject another version of the Gibbs paradox figures more prominently, which deals with the mixing of equal gases only. The paradox now is the fact that a straightforward calculation of the entropy of mixing leads to the previously found quantity $2Nk_{B}\ln2$. This time, however, this value for the entropy increase holds for the mixing of equal gases, contrary to the thermodynamical result according to which entropy remains constant. Note that therefore a solution of this, second, paradox would amount to the restoration of the first paradox.

Both paradoxes apply to classical physics. Nevertheless, in both cases, quantum mechanics has been appealed to for a solution. In case of the original paradox, it is argued that, due to the impossibility of letting the two substances become equal in a continuous process, the paradox disappears, as the following quote from Pauli shows:

“The increase in entropy is always finite, even if the two gases are only infinitesimally different. However, if the two gases are the same, then the change in entropy is zero. Therefore, it is not allowed to let the difference between two gases gradually vanish. (This is important in quantum theory.)”

[2] (p. 48).

In a similar vain, it is often argued that the quantized discreteness of nature explains that there cannot exist an arbitrarily small difference between two substances. In a textbook that still is widely used, Reif writes:

“Just how different must molecules be before they should be considered distinguishable [...]? In a classical view of nature, two molecules could, of course, differ by infinitesimal amounts [...] In a quantum description, this troublesome question does not arise because of the quantized discreteness of nature [...] Hence, the distinction between identical and non-identical molecules is completely unambiguous in a quantum-mechanical description. The Gibbs paradox thus foreshadowed already in the last [i.e., nineteenth] century conceptual difficulties that were resolved satisfactorily only by the advent of quantum mechanics.”

[3] (p. 245).

In case of the second paradox, it is often argued that classical physics gives incorrect results, since it mistakenly takes microstates that differ only by a permutation of particles to be different. Thus, when counting the number of microstates that give rise to the same macrostate, too many microstates are counted in. Thus, for example, in another once popular textbook, Huang writes:

“It is not possible to understand classically why we must divide ∑(E) [the number of states with energy smaller than *E*] by N! to obtain the correct counting of states. The reason is inherently quantum mechanical.”

[4] (p. 141).

Similarly, Schrödinger claims that it is quantum mechanics that solves the Gibbs paradox by pointing out that permutations of like particles should not be counted as different states:

“It was a famous paradox pointed out for the first time by W. Gibbs, that the same increase of entropy must not be taken into account, when the two molecules are of the same gas, although (according to naive gas-theoretical views) diffusion takes place then too, but unnoticeably to us, because all the particles are alike. The modern view (i.e., quantum mechanics) solves this paradox by declaring that, in the second case, there is no real diffusion because exchange between like particles is not a real event—if it were, we should have to take account of it statistically. It has always been believed that Gibbs’s paradox embodied profound thought. That it was intimately linked up with something so important and entirely new (i.e., quantum mechanics) could hardly be foreseen.”

[5] (p. 61).

Yet there is something peculiar in these appeals to quantum mechanics. After all, both versions of the paradox appear in a classical setting. It might very well be that quantum physics gives us a better description of physical reality. However, it would be strange indeed if it were needed to solve puzzles that are interior to classical theories. Rather than turning to quantum mechanics for a solution to the Gibbs paradox, I will argue in this paper that we can learn important lessons by reverting to elementary thermodynamics, and especially to the way in which thermodynamical entropy is intimately related to exchanges of heat, energy, work and particles in reversible processes. The second paradox consists in a difference between thermodynamical and statistical mechanical calculations of the entropy increase when equal gases are mixed. I will show how this difference disappears when the statistical mechanical entropy is introduced in a way that does justice to its thermodynamical origin, by paying close attention to the variation of entropy and the other thermodynamical quantities in reversible processes, rather than simply by counting the number of microstates that lead to the same macrostate.

This paper is structured as follows. In Section 2, I will start by presenting an account of the entropy of mixing that shows how confusions and incorrect results arise when one does not pay close enough attention to the connection between entropy and reversible processes. More specifically, determining the way in which entropy depends on the number of particles by neglecting constants of integration or by fixing these by conventional stipulations could lead to incorrect results. For a correct determination of the entropy difference as a result of a mixing process, one needs to calculate entropy changes during processes in which particle number is actually allowed to vary. Next, I will present three different ways in which one may correctly arrive at the entropy of mixing in thermodynamics. I will briefly indicate why the discontinuous change in the entropy of mixing when gases are considered that are more and more similar should not be seen as paradoxical, whereby the first aspect of the Gibbs paradox is dissolved. In Section 3, I will first discuss the way in which entropy can be introduced in statistical mechanics while being faithful to thermodynamics. I will argue that the Gibbsian framework is much better suited than the Boltzmannian framework to give us proper counterparts of thermodynamical quantities. Moreover, within the Gibbsian framework, it is only the grandcanonical ensemble that is a suitable device for describing processes in which particle numbers vary, such as mixing processes. I will present two different ways in which one could motivate the appearance of the factor 1/N! in the grandcanonical distribution, neither of which makes an appeal to indistinguishability of particles. With the grandcanonical ensemble and this factor of 1/N! in place, one easily recovers the thermodynamical results for the entropy of mixing, both for the mixing of equal and unequal gases, whereby the second aspect of the Gibbs paradox is solved.

## 2. Formulating the Gibbs Paradox in Thermodynamics

Let us start our discussion of the Gibbs paradox in thermodynamics by going over a standard way of deriving the entropy of mixing. This derivation will, surprisingly, turn out to be quite problematic, and the source of confusion. The setup is simple and familiar. We consider the mixing of two equal portions of monatomic ideal gases of equal temperature, volume and number of particles. We want to know the entropy of mixing, that is, the difference between the entropy of the initial state in which the two portions of gas are confined to their own part of the container of volume *V*, and the final state in which the partition is removed and the gas is spread out over the whole container of volume 2V. One arrives at the expression for the entropy of an ideal monatomic gas by starting from the fundamental equation TdS=dU+pdV, and filling in the ideal gas law $pV=Nk_{B}T$ and $U=\left(3/2\right)Nk_{B}T$. We get

(1) $$\begin{array}{ccc} dS & = & \frac{3}{2}Nk_{B}\frac{dT}{T}+Nk_{B}\frac{dV}{V}, \end{array}$$

(2) $$\begin{array}{ccc} S(T,V) & = & \frac{3}{2}Nk_{B}\ln T+Nk_{B}\ln V+c_{1}. \end{array}$$

A straightforward calculation of the entropy of mixing now is

(3) $$\begin{array}{ccc} \Delta S & = & S(T,2V,2N)-2S(T,V,N) \end{array}$$

(4) $$\begin{array}{cc} = & 2Nk_{B}\ln2V-2Nk_{B}\ln V=2Nk_{B}\ln2, \end{array}$$

which gives us the well-known result. So far so good. Or really? We can see that something is not quite in order when we repeat the derivation, but start from expressions for the entropy in terms of a different set of variables. In terms of the pressure *p* and temperature *T*, we have

(5) $$\begin{array}{ccc} S(p,T) & = & \frac{5}{2}Nk_{B}\ln T-Nk_{B}\ln p+c_{2}, \end{array}$$

(6) $$\begin{array}{ccc} \Delta S & = & S(p,T,2N)-2S(p,T,N)=0, \end{array}$$

and if we take the variables to be pressure and volume, we get

(7) $$\begin{array}{ccc} S(p,V) & = & \frac{5}{2}Nk_{B}\ln V+\frac{3}{2}Nk_{B}\ln p+c_{3}, \end{array}$$

(8) $$\begin{array}{ccc} \Delta S & = & S\left(p,2V,2N\right)-2S\left(p,V,N\right)=5Nk_{B}\ln2. \end{array}$$

Clearly, these results contradict each other. What is going on here? Well, in all three derivations, the additive constants $c_{1}$, $c_{2}$ and $c_{3}$ have been set to zero. Since these are constants of integration, they obviously cannot depend on the variables, but they may still depend on anything else—including the number of particles! The above expressions for the entropy simply do not fully specify the way in which entropy varies with particle number. They treat particle number as a parameter, not as a variable. In fact, one easily checks that

(9) $$c_{1}=c_{2}-Nk_{B}\ln Nk_{B}=c_{3}+\frac{3}{2}Nk_{B}\ln Nk_{B},$$

and so it is clear that setting all three constants to zero leads to incompatible results. In fact, these constants may still depend on the number of particles, and it turns out that they are *different* functions of particle number. Setting either one of the constants $c_{1}$, $c_{2}$ and $c_{3}$ to zero is thus a conventional choice that stands in need of justification.

There is another peculiar feature of the above derivations. Have we attempted to derive the entropy change when two different ideal gases were mixed, or two portions of the same gas? No assumption as to the nature of the ideal gas went into the derivations. One way of looking at this is to note that the additive constants may not only still depend on the particle number, but also on the kinds of gases involved. Somehow, the derivation with $c_{1}=0$ leads us to the correct result for the mixing of two different gases, and the derivation with $c_{2}=0$ leads us to the correct result for the mixing of two portions of the same gas. This, however, so far seems to be just a coincidence. We need to have a better look at thermodynamical theory, and redo the derivation of the entropy of mixing.

Let us go back to the basics (for a more detailed account, I refer to [6]). Entropy is introduced into the orthodox theory of thermodynamics as the state variable one gets when integrating δQ/T along a curve in equilibrium space:

(10) $$\int_{A}^{B}\frac{\delta Q}{T}=S_{B}-S_{A}.$$

Here, δQ is the amount of heat that is exchanged in an infinitesimal process. It is an inexact differential, and *Q* is not a state variable, i.e., it cannot be expressed as a function on equilibrium space. The differential dS on the other hand is exact, and *S* is a state variable. By definition Equation (10), only entropy *differences* are defined, that is, entropy has been defined up to a constant of integration. Moreover, only entropy differences are defined between equilibrium states that can be connected by a process that fully takes place in equilibrium space, i.e., by a quasistatic process. This means, for example, that entropy values for non-equilibrium states so far have not been defined. Neither are entropy differences defined between, say, one mole of oxygen and one mole of argon, since these cannot be connected by a quasistatic process.

There are various ways in which one may extend the definition of entropy. One may, for example, fix the integration constant by reference to a fiducial state. This, however, will not help us out in the case of the Gibbs paradox, since this method does not work for comparing entropy values of different kinds of gas, again since these cannot be connected by a quasistatic process. Another common convention is to appeal to the third law of thermodynamics, which states that all entropy differences (as far as they are defined!) approach zero when the absolute temperature approaches zero. This invites the conventional choice of setting not only all entropy differences but also all entropy values to zero in this limit. Unfortunately, this again will not be a convenient choice for the setting of the Gibbs paradox, since classical ideal gases do not obey the third law. Another conventional choice is to take the entropy to be *extensive*, that is, require that the entropy increases by a factor of *q* when the system as a whole increases by *q*. Note that this is a rough characterisation, since it is not always clear what it means for a system to increase by a certain factor, especially not for inhomogeneous systems. However, it is sufficiently clear in cases where entropy is given as a function of other state variables that themselves are clearly intensive (such as temperature and pressure) or extensive (such as volume and number of particles). We may then require, say, that S(T,qV,qN)=qS(T,V,N). Note, incidentally, that this requirement immediately yields ΔS=0 for the entropy difference in Equation (3). However, another extension of the definition of entropy is to require *additivity*, that is, take the entropy of a composite system to be the sum of the entropies of the constituents, also when those constituents are not in equilibrium with one another. (Note that additivity indeed differs from extensivity. The composite system one gets when combining two containers of volume *V* still includes the walls of both containers. Extensivity, on the other hand, applies to extending systems without placing walls between subsystems.) This opens up the whole field of non-equilibrium thermodynamics.

However, in extending the definition of entropy, one needs to proceed with care. Some extensions, such as additivity, simply enlarge the applicability of the notion. However, others bear the risk of fixing entropy differences or absolute values that are *already* fixed by the definition Equation (10). Thus, what about extensivity of entropy? Can this safely be assumed? In many cases, it is simply stated that entropy in thermodynamics clearly *is* extensive (see for example [7,8]). Based on definition Equation (10), this cannot be quite right, since that definition does not determine entropy values, only differences, so that the issue of extensivity is underdetermined. A more careful claim would be to state that extending the definition of entropy by requiring extensivity does not lead to conflict with the rest of thermodynamics. However, one may wonder whether this is actually the case. One interesting account in this respect is given by Landsberg [9], who turns the claim that it is possible to consider all of the most important thermodynamical quantitites as either intensive or extensive into a fourth law of thermodynamics. This, in my view, only highlights the conceptual possibility that entropy is *not* extensive. Thus, the most careful thing to do here is to refrain from extending the definition of entropy, and to make use of Equation (10) in order to calculate entropy differences along quasistatic processes. Fixing the integration constants by conventions would run the risk of introducing conflicts with entropy differences that are *already* determined.

Let us return to the entropy of mixing. If we want to improve on the derivations given above, we should not make unwarranted assumptions about the *N*-dependence of the entropy. A parsimonious derivation is to be preferred over one that makes abundant use of extra assumptions on top of the original definition of entropy differences, and of course conflicting assumptions such as setting all three constants $c_{1}$, $c_{2}$ and $c_{3}$ to zero should be avoided at all cost. A further desideratum is that it should be clear from the derivation whether it applies to the mixing of equal or different gases. Fortunately, several such derivations are available, which however interestingly differ with respect to the exact assumptions that are appealed to. I will discuss three of them in turn.

The most straightforward thing to do is to make use of expressions for the entropy that truly treat particle number as a variable rather than as a parameter. That is, we add to the fundamental equation terms that take into account varying particle numbers:

(11) $$TdS=dU+pdV+\sum_{i}\mu_{i}dN_{i}$$

so that it becomes possible to calculate the way in which entropy varies with varying number of particles of kind *i*. We follow a derivation given by Denbigh [10] (pp. 111–118), which applies to mixtures of perfect gases. A perfect gas is more general than an ideal gas, since the specific heat may be an arbitrary function of the temperature, rather than a constant $\left(3/2\right)Nk_{B}$. Denbigh defines a perfect gas mixture as one in which each component satisfies

(12) $$\mu_{i}\left(p,T,\left\{N_{i}\right\}\right)=\mu_{i}^{0}\left(T\right)+k_{B}T\ln\left(\frac{N_{i}}{\sum_{i}N_{i}}p\right).$$

Denbigh further assumes both additivity and extensivity of entropy, and calculates the entropy $S(T,V,N_{A},N_{B})$. It then straightforwardly follows that when two different gases are mixed, the entropy increases by

(13) $$\begin{array}{ccc} \Delta S & = & S\left(T,2V,N_{A},N_{B}\right)-\left(S\left(T,V,N_{A}\right)+S\left(T,V,N_{B}\right)\right) \end{array}$$

(14) $$\begin{array}{cc} = & \left(N_{A}+N_{B}\right)k_{B}\ln2, \end{array}$$

and when two equal gases are mixed, the entropy remains constant. We thus have the familiar results, on the basis of assumptions of additivity and extensivity of entropy and a definition of the chemical potential of perfect gases. How about the difference between the cases of mixing different or equal gases? One could say that this difference is introduced by definition: for each kind of gas, a term $\mu_{i}dN_{i}$ is included in the fundamental equation. Suppose we decided to treat a pure gas as a mixture of two portions of the same kind of gas, and added a term $\mu_{i}dN_{i}$ for each of those portions. We then would also find the entropy increase we found in Equation (14) in the case of mixing the same gas! This leaves us with a somewhat unsatisfactory treatment of the difference between the two cases.

Another derivation of the entropy of mixing has been given by Planck [11]; it is both sparser in its assumptions, and clearer on the distinction between mixing different or equal gases. Planck does not fully calculate the entropy as a function $S(T,V,N_{A},N_{B})$ of the state parameters. Instead, he directly calculates entropy differences along quasistatic processes only for the mixing processes of interest. For this, he uses a construction with semi-permeable membranes, which allow particles of one kind to go through. Suppose we start with a container of volume *V* that contains a mixture of two gases *A* and *B*, and with two membranes: one on the far left side that lets through only particles of kind *A*, and one on the far right side that lets through only particles of kind *B*. These containers can now be slowly extended like a telescope, leaving us in the end with two containers of volume *V* each, where the left container is filled by gas *A* and the right container is filled by gas *B*. Suppose further that Dalton’s law holds, that is, the pressure of a gas equals the sum of partial pressures. Then, it follows that, during the extension, no work is done, and moreover, the total energy and amount of particles remain constant. This extension therefore leaves the entropy constant. Next, the volume of each container is compressed in a quasistatic, isothermal process to V/2, resulting in an entropy change

(15) $$\Delta S=\int_{V}^{V/2}\frac{\delta Q}{T}=-\int_{V}^{V/2}Nk_{B}\frac{dV}{V}=-Nk_{B}\ln2$$

for each of the two containers. Now, considering the whole process in reverse so that we start with the separate gases and end with the mixture, we arrive at an entropy increase of $2Nk_{B}\ln2$. It is immediately clear that this construction does not apply to the mixing of two portions of the same gas. After all, a semi-permeable membrane that is transparent to gas *A* but not to gas *A* cannot exist. For the mixing of equal gases, we simply appeal to the reasoning given earlier, namely that we can restore the initial thermodynamical state simply by putting back the partition, at no entropy cost. Therefore, there is no entropy of mixing in this case.

A third derivation of the entropy of mixing has been given in a wonderful paper by Van Kampen [12]. Now, however, the entropy of mixing is understood to be the absolute value of the entropy of a mixture, not the entropy difference as the result of a mixing process. Van Kampen is careful and explicit in formulating conventions with respect to the entropy value, and takes great care not to compare entropy values “belonging to different *N*, unless one introduces a new kind of process by which *N* can be varied in a reversible way” [12] (p. 305). The first convention he appeals to is to take the integration constants equal for systems that are identical. The second convention is the additivity of entropy. These conventions make it possible to derive an expression for entropy that still contains an integration constant, but for which the dependence on particle number has been fixed. For a single gas, the procedure is simply to remove a partition between containers with different portions of that single gas. Van Kampen finds

(16) $$S\left(p,T,N\right)=\frac{5}{2}Nk_{B}\ln T-Nk_{B}\ln p+cN.$$

For a mixture, the procedure is to mix or separate the gases by making use of semi-permeable membranes. On this basis, Van Kampen finds

(17) $$S\left(p,T,N_{A},N_{B}\right)=\frac{5}{2}\left(N_{A}+N_{B}\right)k_{B}\ln T-Nk_{B}\ln p_{A}^{\prime }-Nk_{B}\ln p_{B}^{\prime }+c_{A}N_{A}+c_{B}N_{B},$$

where also Dalton’s law has been used. One notes that the entropy of a mixture in Equation (17) does not reduce to the entropy of a pure gas in Equation (16) when *A* and *B* are the same, and $N_{A}+N_{B}=N$. This, according to Van Kampen, is the Gibbs paradox. There is an interesting parallel with the remark we made above about the entropy of mixing in Equation (14), where the value of the entropy of mixing also seemed to depend on whether we choose to consider a gas as consisting of one single gas *A*, or of a mixtures of two portions of that very gas. Van Kampen, however, unlike Denbigh, treats the difference between mixtures of different or equal gases with great care. He extends the definition of entropy by an appeal to reversible processes that connect systems of different particle number. The process for doing so in the case of equal gases, namely the removal or addition of a partition, clearly cannot be used for reversibly mixing or separating different gases. Conversely, mixing or separating gases by means of semi-permeable membranes is applicable to the case of unlike gases only. Thus, where Denbigh’s derivation introduces the distinction by definition, Van Kampen gives us an explanation of this distinction. How does Van Kampen’s treatment of the Gibbs paradox fare with respect to our other desideratum, parsimony of assumptions? In this respect, Planck’s treatment is still superior. Both assume Dalton’s law, and appeal to semi-permeable membranes that can be used to separate the two kinds of gas. However, on top of this, Van Kampen makes use of two conventions that fix entropy values, rather than just entropy differences in a mixing process. Planck’s derivation shows that this is not necessary in order to derive the difference between mixing the same or different gases, and thus in order to arrive at the Gibbs paradox. The core of the original, thermodynamical paradox thus lies in the procedures by which the definition of entropy *differences* can be extended to cases in which particle number varies. It does not lie in the conventions that can be used in order to fix the *value* of entropy.

About solutions to the original, thermodynamical paradox, I want to be brief. The modern standard response is that there is indeed a discontinuity between mixing the same or different gases, but that there is nothing remarkable about that [12,13]. The construction with the semi-permeable membranes shows that differences between the two cases should not surprise us. Some authors [14] further motivate this viewpoint by an appeal to a subjective interpretation of entropy, according to which entropy measures the amount of information that is available to a subject. On such a view, it is up to the experimenter to regard two gases either as equal or as different, and the entropy of mixing depends on this subjective choice. Other authors [15] further motivate the viewpoint by an appeal to an operationalist approach to thermodynamics, according to which the meaning of thermodynamical notions is given by a set of operations (which may be either physical, or “pencil and paper operations”). However, since the operations that define the entropy of mixing of two portions of the same gas differ from those of mixing different gases, a discontinuous change in the entropy of mixing is not considered to be remarkable. We may, however, abstract away from these two particular motivations. One need not be committed to either subjective approaches to statistical physics or to operationalism to appreciate the general point that there is nothing paradoxical about the discontinuity.

The lessons that we should learn from the thermodynamical paradox do not concern the solution to the original paradox, but rather the way in which entropy differences are tied to reversible processes, and the question of whether the definition of entropy needs to be extended by conventions such as extensivity. Jaynes [14] makes interesting observations about this in a discussion of Pauli’s account (which is similar to Van Kampen’s account discussed above), who requires extensivity in order to fix entropy values. Jaynes writes:

“Note that the Pauli analysis *has not demonstrated from the principles of physics that entropy actually should be extensive*; it has only indicated the form our equations must take if it is. However, this leaves open two possibilities:

1. All this is tempest in a teapot; the Clausius definition (i.e., dS=δQ/T) indicates that only entropy differences are physically meaningful, so we are free to define the arbitrary additive terms in any way we please. […]
2. The variation of entropy with *N* is not arbitrary; it is a substantive matter with experimental consequences. Therefore, *the Clausius definition of entropy is logically incomplete*, and it needs to be supplemented either by experimental evidence or further theoretical considerations.”

Neither option is exactly right, I would say. We are *not* free to define the additive terms however we like, since fixing them by convention may easily lead to confusion or even erroneous results, as we have seen. It would lead to two kinds of entropy differences: those to which the Clausius definition applies, and those that are fixed by conventions. The incorrect impression may arise that also entropy differences that have been introduced by convention correspond to heat exchanges in quasistatic processes. This, however, is generally incorrect, since the additive constants in this way are also determined for entropy differences between states that cannot be connected by physical processes, such as one mole of oxygen and one mole of argon. Errors may result in cases where the additive constants are determined by convention, and where also quasistatic processes are possible by means of which the entropy differences can be determined. We have seen examples of this in the above derivations of Equations (4) and (8). The second option Jaynes presents suggests that Clausius’ definition is insufficient to describe processes in which particle number varies. However, this is also incorrect: by considering quasistatic processes in which particle number is allowed to vary, entropy differences can be determined.

My own conclusion would be that the Clausius definition suffices to describe thermodynamical processes. The additive constants should not be neglected, and can still depend on anything but the state parameters of which entropy is a function. Extending the definition of entropy to absolute entropy values is not necessary, and is moreover undesirable, since fixing the constants may lead to confusion and error.

## 3. Formulating the Gibbs Paradox in Statistical Mechanics

### 3.1. Entropy in Statistical Mechanics

In statistical mechanics, discussions about the Gibbs paradox typically center around the correct way of counting microstates. We now encounter a second version of the paradox, which only deals with mixing two portions of the same gas. Standard calculations lead to an entropy increase by the amount of $2Nk_{B}\ln2$ also in the case of mixing the same gas, which is in conflict with the thermodynamical result. A standard response is that the way in which the number of microstates that correspond to a given macrostate is calculated, should be modified, in order to arrive at the desired result that the entropy does not increase when two portions of the same gas are mixed. Thus, this correction re-introduces the distinction between mixing of equal and different gases, and re-introduces the first version of the paradox.

Here is one such standard calculation of the entropy of mixing, working from the Boltzmannian framework to statistical mechanics (see, for example, [7,8,16]). The Boltzmann entropy is given by $S_{B}=k_{B}\ln W$, where *W* counts the number of microstates corresponding to a certain macrostate. Now, suppose we have an ideal gas, consisting of *N* molecules that each can be in any of *X* possible states. We then find that $W=X^{N}$, and thus $S=Nk_{B}\ln X$. A moment’s reflection shows that this function is *not* extensive: when the gas doubles in size, not only the number of particles, but also the available volume in phase space per particle doubles. We again find an entropy of mixing by the amount of $2Nk_{B}\ln2$. For another standard calculation (see, for example, [3,17]), we find in the Gibbsian framework that the entropy of an ideal canonically distributed gas leads us back to the thermodynamical relation Equation (2) with $c_{1}=0$, and so, again, to the entropy of mixing we also found in Equation (4).

It is time to take the lessons from the previous section to heart, and have a more careful look. Neither the Boltzmann entropy, nor the Gibbs entropy of the canonical ensemble, tell us how entropy varies in processes in which the number of particles is allowed to vary. They treat particle number as a *parameter*, not as a *variable*. Moreover, the calculations we just gave neglect the additive constants, and do not pay attention to the fact that these may still depend on particle number. Again, in order to do better, we need to attend to reversible processes in which particle number is allowed to vary. It is the Gibbs formalism that offers a way to do so, and more specifically not the canonical but the grandcanonical ensemble.

The standard counterpart of thermodynamical entropy within Gibbsian statistical mechanics is the so-called fine-grained entropy, or Gibbs entropy. It is defined not as a function on phase space, but as a functional on a probability distribution ρ(x) over points *x* in phase space:

(18) $$S_{fg}\left[\rho \right]=-k_{B}\int \rho \left(x\right)\ln\rho \left(x\right)dx.$$

Gibbs himself was reluctant to view Equation (18) as the counterpart of thermodynamical entropy for general probability distributions ρ(x). He showed only for special choices of the probability distributions, namely the microcanonical, canonical and grandcanonical ensembles, that the fine-grained entropy shares a number of properties with thermodynamical entropy, notably, that it obeys the fundamental equation TdS=δQ=dU−δW. In fact, for the canonical ensembles, one can do a bit more than this, and show that the fine-grained entropy is the *unique* function that obeys this fundamental equation (see, for example, [18] (pp. 21–24)). In order to do so, identifications need to be made for all terms in the fundamental equation. Generally, the statistical mechanical counterparts of thermodynamical quantities are not taken to be the functions of phase space, with all their microscopic detail and variability. Rather, they are taken to be either expectation values of phase functions, or parameters of the probability distribution, or some combination of those. In this vain, the statistical mechanical counterpart of *U* is straightforwardly taken to be the expectation value of the Hamiltonian ${\langle H\rangle }_{\rho }$. The work performed by the system δW is identified with

(19) $$\delta W=\sum_{k}{\langle A_{k}\rangle }_{\rho }da_{k}=-\sum_{k}{\langle \frac{\partial H}{\partial a_{k}}\rangle }_{\rho }da_{k},$$

where the $a_{k}$ denote external parameters such as the volume. Remember that in thermodynamics the relation TdS=δQ holds for “infinitely slow” processes. The crucial point is that these are now construed as processes in which the system remains a member of a Gibbsian ensemble. Uhlenbeck and Ford write:

“We will now show that for a change δ in which *both* the β of the heat reservoir *and* the parameters $a_{k}$ are changes in such a slow or “reversible” way that the system may always be considered to be canonically distributed, the quantity βδQ is a perfect differential of a function of the state of the system, that is a function of β and the $a_{k}$.”

[18] (p. 21).

That is, it is assumed that if a system is (micro-; grand-)canonically distributed, then, after small changes in the state parameters, the system will again be member of a (micro-; grand-) canonical ensemble, albeit a different one. In this respect, the system is in equilibrium all the time during these processes. (It is important to note that this kind of “infinitely slow” processes are not allowed by the dynamics. That is, a curve consisting of (micro-; grand-) canonical distributions only cannot be the solution of the Liouville equation, not even with a time-dependent Hamiltonian. For a modification of the derivation by Uhlenbeck and Ford that also applies to dynamically allowed processes, see [19]). For the canonical ensemble, this procedure leads to

(20) $$S\left(T,\left\{a_{k}\right\}\right)=\frac{\partial }{\partial T}\left(k_{B}T\ln Z\right)+c_{0},$$

where $c_{0}$ is an integration constant, and *Z* is the canonical partition function

(21) $$Z\left(\beta ,\left\{a_{k}\right\}\right)=f\left(N\right)\int e^{-\beta H(x,\left\{a_{k}\right\})}dx,$$

where $\beta =1/(k_{B}T)$ and where f(N) is a function of *N* that we presently take to be arbitrary, but that is often taken to be 1/N! (more on this below).

In order to apply the same procedure to the grandcanonical ensemble, we note that the grandcanonical partition function

(22) $$Z_{g}=\sum_{N_{1},\ldots ,N_{n}}f\left(N_{1},\cdots ,N_{n}\right)e^{\left(\sum_{k}\alpha_{k}N_{k}\right)}\int e^{-\beta H(x,\left\{a_{k}\right\})}dx$$

allows us to write

(23) $$\bar{N_{i}}={\left(\frac{\partial }{\partial \alpha_{i}}\ln Z_{g}\right)}_{\beta ,\left\{\alpha_{j}\right\},\left\{a_{k}\right\}}\left(i\neq j\right).$$

Here, for convenience, we have written α=βμ. We now add a term $\mu_{i}d\bar{N_{i}}$ to the fundamental equation. That is, we take as the counterpart of the thermodynamical number of particles the grandcanonical expectation value of *N*, whereas we identify the chemical potential μ with a parameter in the probability distribution, in parallel to the temperature parameter β. With these identifications in place, we find the following expression for the grandcanonical entropy:

(24) $$S_{g}\left(\left\{\mu_{k}\right\},T,\left\{a_{k}\right\}\right)=\frac{\partial }{\partial T}\left(kT\ln Z_{g}\right)+c_{1}.$$

This is strikingly similar to Equation (20) but differs in an important respect for present purposes: it specifies how entropy depends on particle number, since the (expected) numbers of particles $\bar{N_{i}}$ are functions of the state parameters $\left\{\mu_{k}\right\}$, *T* and $\left\{a_{k}\right\}$. Thus, the integration constant $c_{0}$ in Equation (20) may still depend on particle number, but the constant $c_{1}$ in Equation (24) may not.

We are now in the position to clear up some of the confusion related to attempts to solve the Gibbs paradox by fixing the *N*-dependence of the canonical partition function, that is, by specifying f(N). From Equation (20), we see that

(25) $$S\left(T,\left\{a_{k}\right\}\right)=\frac{\partial }{\partial T}\left(k_{B}T\ln\int e^{-\beta H(x,\left\{a_{k}\right\})}dx\right)+k_{B}\ln f\left(N\right)+c_{0},$$

where, as noted before, the constant may still depend on *N*. We see immediately that fixing the factor f(N) does not fix the *N*-dependence of the canonical entropy, since any change in this factor may be counterbalanced by the integration constant $c_{0}$. In addition, the factor f(N) does not affect any expectation values of phase functions, since it cancels out by normalization.

For the grandcanonical ensemble, however, things are different. This should not surprise us, since the grandcanonical ensemble applies to systems of which particle number is allowed to vary. Indeed, the factor $f(N_{1},\cdots ,N_{n})$ in the partition function now affects both the value of the grandcanonical entropy Equation (24), and also the expectation values of phase functions. Thus, it is only in the case of the grandcanonical ensemble that fixing the *N*-dependence becomes important. In the following subsection, we will turn to arguments for fixing this *N*-dependence.

### 3.2. N!

Arguments for adding a factor 1/N! to the partition function roughly fall into two categories. First, one may appeal to the correct way in which microstates need to be counted. Secondly, one may assume extensivity of entropy (or, as we shall see, the intensivity or extensivity of related quantities). I will discuss several arguments in turn.

First and foremost, the factor 1/N! is often motivated by the claim that already in the classical domain microstates that only differ by a permutation of particles of the same kind should be treated as the same microstate (see, for example, [3,4,5,17,20,21]). The factor enters the partition function as a correction by which one divides by the number of ways in which *N* particles can be permuted. This correction is often motivated by an appeal to quantummechanical indistinguishability. That this shows up already in the classical domain is then seen as a remarkable and profound result—witness the quote from Schrödinger in the introduction. Saunders [21] gives a different twist to the story, by arguing that classical indistinguishability of particles is not mysterious at all. He writes: “Why not treat permutations just like any other symmetry group, and factor them out accordingly?” This kind of reasoning relates back to a discussion between on the one hand Planck [20], and, on the other hand, Ehrenfest and Trkal [22] in the 1920s, so even before the rise of quantum mechanics. Here, Saunders’ viewpoint is in line with Planck, who claims that the statistical method does not require one to count the number of states in which *particles* can be, but in which the *gas as a whole* can be. In addition, for the gas as a whole, it does not matter which particle is in which state, since all particles are the same. Ehrenfest and Trkal counter that it is the core of the method of statistical physics that one determines the number of microstates that lead to states that are macroscopically indistinguishable. Permutations of like particles result in states that differ from each other at the microlevel, and therefore should not be treated as the same microstate. Indeed, when truly counting in all microstates that result in the same macrostate and refraining from factoring out symmetries, one stays closer to the spirit of statistical physics (see, for example, [8,12,13,16] for a similar diagnosis).

A completely different argument in favour of adding 1/N! to the partition function is given by Van Kampen [12], who in turn makes use of a more general argument given by Ehrenfest and Trkal [22]. Van Kampen shows how this factor in the grandcanonical distribution can be derived by taking a suitable limit from the canonical distribution. Van Kampen considers a gas consisting of *N* particles in volume *V* and with Hamiltonian H(x) that is in contact with a gas consisting of $N^{\prime }$ particles in volume $V^{\prime }$ and with Hamiltonian $H^{\prime }\left(x^{\prime }\right)$. The combined system is canonically distributed, and we write $N^{*}=N+N^{\prime }$, $V^{*}=V+V^{\prime }$ and $H_{N^{*}}^{*}=H_{N}+H_{N^{\prime }}^{\prime }$. He calculates the probability W(N,x) to find *N* random particles in *V* in the point in phase space *x*. This requires, first, to select *N* particles from amongst the total amount of $N^{*}$ particles. Since the other $N^{\prime }$ particles may be in an arbitrary state, we integrate and find

(26) W(N,x)=cN*Ne−βHN(x)∫e−βHN′′(x′)dx′,

where the normalization constant is found by intregrating over all possible states of the combined system:

(27) $$c^{-1}=\int e^{-\beta H_{N^{*}}^{*}\left(x^{*}\right)}dx^{*}.$$

When we now take the limit $N^{*}\to \infty$, $V^{\prime }\to \infty$ at a constant density $\rho \equiv N^{*}/V^{\prime }$, the system in volume $V^{\prime }$ can be regarded as a particle reservoir. In this limit, one finds

(28) $$W\left(N,x\right)=c\frac{z^{N}}{N!}e^{-\beta H_{N}\left(x\right)},$$

where *z* is a function of ρ and β. This is the grandcanonical distribution, and it contains the factor 1/N!. A similar calculation applies to systems containing several kinds of particles. The construction now requires several particle reservoirs, each of which is connected by a semi-permeable membrane to the system of interest. This results, as one may expect, in factors $1/N_{i}!$ for each of the kinds of particles.

It is a remarkable fact that, in Van Kampen’s construction, it is not the *indistinguishability* of classical particles that gives rise to the factor 1/N!. Rather, it is the *distinguishability* of classical particles! Van Kampen does not *divide* by the number of microstates that lead to the same macrostate, but rather *multiplies* by the number of possibilities to choose the *N* particles from among the total amount of $N^{*}$. Thus, all microstates that lead to the same macrostate are taken into account; states that only differ by a permutation of the particles are all counted separately. Still, in the suitable limit, one finds that 1/N! appears in the grandcanonical distribution function.

A different argument for adding 1/N! to the partition function has been given in a short and barely noticed paper by Buchdahl [23]. He presents his argument as a classical argument, in which considerations of quantum mechanical indistinguishability play no role, and in which no convention is required about extensivity of entropy. Rather, he claims that internal consistency of the Gibbsian ensemble formalism demands that 1/N! should be present in both the canonical and grandcanonical partition function. Below, I will criticize parts of his argumentation, but what will survive my criticism is still an interesting conclusion: for the grandcanonical partition function (though not for the canonical partition function), 1/N! can be derived on the basis of this classical argument. Extensivity of entropy is however also required in order to reach this conclusion.

By internal consistency of the Gibbs formalism, Buchdahl means that the canonical and grandcanonical partition functions ought to depend in the same way on the number of particles. That is, the grandcanonical ensemble should be a weighted sum of the canonical ensemble

(29) $$Z_{g}=\sum_{N=0}^{\infty }\lambda^{N}Z_{N}=\sum_{N=0}^{\infty }\lambda^{N}g_{N}\int e^{-\beta H(x)}dx.$$

Here, $g_{N}$ is the *N*-dependence of the partition function that this argument aims to determine, and λ=exp(βμ). Buchdahl now appeals to the following relations for the thermodynamic potential *X*:

(30) $$\begin{array}{ccc} X & = & pV=k_{B}\ln Z_{g}, \end{array}$$

(31) $$\begin{array}{ccc} \frac{\delta X}{\delta V} & = & \frac{X}{V}. \end{array}$$

From this, it follows that

(32) $$Z_{g}=ae^{bV},$$

where *a* and *b* are functions of *T* and μ alone. In order to determine $g_{N}$ it suffices to compare Equations (29) and (32) for a specific case, namely the ideal gas, for which $Z_{g}^{id}=\sum \lambda^{N}g_{N}{\left(V/\lambda_{T}^{3}\right)}^{N}$. One finds that

(33) $$g_{N}=a{\left(b/\tau \lambda \right)}^{N}/N!,$$

where *a* and b/τλ need to be constant, so that we end up with $g_{N}=1/N!$.

As remarked earlier, Buchdahl presents this argument as an alternative to requiring extensivity of entropy. However, in fact, such a requirement is hiding behind Equation (30)! Buchdahl refers for this relation to his textbook [24], and it turns out that he there derives this relation on the basis of the assumptions of extensivity of entropy *S*, extensivity of energy *U*, and extensivity of the thermodynamic potential *X*. Now, as a matter of fact, one could be a little sparser in the assumptions and still arrive at Equation (30); this relation also follows from assuming extensivity of the volume *V* and intensivity of the chemical potential μ. (This follows by applying Euler’s theorem to the chemical potential X(T,V,μ)). However, since intensivity of μ and extensitivity of *S* are intimately related through

(34) $$\mu \left(E,V,N\right)=-T{\left(\frac{\partial S}{\partial N}\right)}_{E,V},$$

these assumptions are of a similar kind. My other point of criticism is that the requirement expressed by Equation (29) is too strict. Why would internal consistency of the Gibbs formalism demand that the *N*-dependence of the canonical and grandcanonical ensembles is exactly the same? After all, variation of particle number exactly marks the difference between the two ensembles. In my view, it would be more reasonable to demand that the grandcanonical partition function is indeed a weighted sum of canonical partition functions, but with weights that are allowed to vary with *N*:

(35) $$Z_{g}=\sum_{N=0}^{\infty }f_{N}\lambda^{N}Z_{N}.$$

The previous argument then only determines the product $f_{N}g_{N}$, and thus only fixes the *N*-dependence of the grandcanonical ensemble, not of the canonical ensemble. However, note that this is all that we were after. As I argued earlier, the *N*-dependence in the canonical ensemble is immaterial, since it does not affect the value of entropy differences or of ensemble averages. Thus, Buchdahl does provide us with a classical account of the *N*-dependence in the case where it actually matters.

Let us briefly compare the assumptions underlying the two ways in which we have determined the *N*-dependence in the grandcanonical partition function. Both in their own way are concerned with an internal consistency of the Gibbs formalism. In Van Kampen’s case, the grandcanonical distribution is derived from the canonical distribution in a suitable limit, in which part of the canonically distributed system is playing the role of a particle reservoir for the rest of the system. In case of systems containing several kinds of particles, this construction makes use of semi-permeable membranes. In the modified Buchdahl case, the requirement of internal consistency is that the grandcanonical partition function is a weighted sum of canonical partition functions. On top of this, the modified Buchdahl case also requires either the assumption that entropy is extensive, or the assumption that the chemical potential is intensive. It is clear that Van Kampen’s derivation is the more parsimonious one.

Saunders comments as follows on Van Kampen’s derivation:

“Why so much work for so little reward? Why not simply assume that the classical description be permutable (i.e., that points of phase space related by a permutation of all $N^{*}$ particles represent the same physical situation)?”

[21] (p. 196).

I respond by noting that the reward should not be underestimated. What Van Kampen delivers is a proper justification for adding the 1/N! to the grandcanonical partition function that stays true to the core of classical statistical mechanics by taking into account all microscopic configurations that in principle could be distinguished. Combining his argument with Uhlenbeck and Ford’s way of relating the grandcanonical entropy from Equation (24) to quasistatic changes in other thermodynamical quantities yields a thoroughly justified statistical mechanical account of processes in which particle number is allowed to vary.

### 3.3. The Gibbs Paradox in Statistical Mechanics

We finally turn to the entropy of mixing in a statistical mechanical description of the mixing process. Since we are dealing with processes in which the particle number varies, we appeal to the grandcanonical ensemble. Fortunately, the *N*-dependence of the partition function and thus also of the entropy have now been determined. We can simply calculate the entropy of an ideal gas by plugging the partition function $Z_{g}^{id}=\sum \left(\lambda^{N}/N!\right){\left(V/\lambda_{T}^{3}\right)}^{N}$ into Equation (24), and we find

(36) $$S_{id}\left(T,V,\left\{\bar{N_{i}}\right\}\right)=\sum_{i}k_{B}\bar{N_{i}}\left(5/2+\ln\frac{V}{\bar{N_{i}}\lambda_{T}^{3}}\right)+c_{1}.$$

For the mixing of two equal gases, we find

(37) $$\Delta S=S\left(T,2V,2\bar{N}\right)-2S\left(T,V,\bar{N}\right)=0$$

and, for the mixing of two different gases, we find

(38) $$\Delta S=S\left(T,2V,\bar{N_{A}},\bar{N_{B}}\right)-(S\left(T,V,\bar{N_{A}},0\right)+S\left(T,V,0,\bar{N_{B}}\right)=\left(\bar{N_{A}}+\bar{N_{B}}\right)k_{B}\ln2.$$

We thus arrive at the same results as in thermodynamics. This means that we have solved the second version of the Gibbs paradox, by restoring the original Gibbs paradox. For just the same reasons as in the thermodynamical case, the discontinuous difference between mixing the same or different gases should not be considered paradoxical.

In Section 2, we discussed *three* different ways of correctly arriving at the entropy of mixing. In fact, we can reproduce all of them in the statistical mechanical context. The calculation just presented parallels Denbigh’s derivation of Equation (14), even though an important difference is that now we did not have to put in as an assumption that entropy is extensive. Van Kampen’s account of the Gibbs paradox can also be parallelled in the context of the grandcanonical ensemble. He himself now does not calculate the value of the entropy of mixing, but already finds paradox in the grandcanonical probability function. The probability function for a mixture of two gases is given by

(39) $$W\left(N_{A},N_{B},x_{A},x_{B}\right)=c\frac{z_{A}^{N_{A}}z_{B}^{N_{B}}}{N_{A}!N_{B}!}e^{-\beta H_{N_{A},N_{B}}\left(x_{A},x_{B}\right),}$$

and this does not reduce to Equation (28) when *A* and *B* are equal, and $N=N_{A}+N_{B}$. Finally, we could also simply copy Planck’s line of reasoning, and conclude that separating a mixture by means of his telescopic construction with semi-permeable membranes leaves the entropy constant. We then again only need to compute the entropy change for an isothermal compression of the two containers, each containing a single gas. Completely analogous to Equation (15), we again arrive at the same result. This time, however, we might as well work with the canonical rather than the grandcanonical ensemble, since the number of particles remains constant during the compression.

Statistical mechanics is a far richer theory than thermodynamics because it brings into the picture the details of microscopic motion, and the interactions between molecules. Such details matter also in the description of mixing processes: arbitrary Hamiltonians can be taken into account, and fluctuations can be computed. However, even though microscopic details play their role and have their influence on the value of entropy differences, entropy also in the statistical mechanical context is a function of just a handful of macroscopic variables. Stripped down to the simple case of classical ideal monatomic gases, the functional relationships between entropy, volume, temperature and particle number are the same as in thermodynamics. We thus find the same results for the entropy of mixing as we did in thermodynamics.

Let me end by briefly commenting on yet another twist in the rich stories about the Gibbs paradox. Dieks [13] (p. 371) claims that ‘if the submicroscopic particle picture of statistical mechanics is taken completely seriously, the original formula S=klogW, without the ad hoc division by N!, gives us correct results’—for we may imagine there to be semi-permeable membranes that are not only sensitive to the *kinds* of gas *A* and *B*, but also to individual particle trajectories and origins. Such membranes can be used to bring back all particles to their original container also after two portions of the same gas have been mixed. Thus, in parallel to Planck’s reasoning, we would also find an entropy increase when mixing the same gas. Moreover, this is now seen as the correct statistical mechanical result. Dieks claims that the practical issue that such membranes do not exist and macroscopic separation techniques will not be capable of selecting particles on the basis of their individual trajectories is irrelevant to the more principled issue of whether particles are distinguishable. To this much I agree; also in the original construction given by Planck, the membranes need not actually be available in practice in order for them to do their conceptual work in shedding light on the difference between mixing equal or unequal gases. Indeed, when one views the final state after mixing equal gases as different from the initial state because not exactly the same particles are located in each of the containers, then obviously work needs to be done in order to restore the initial state, and there is an entropy of mixing also in the case of mixing the same gas. However, this does not mean that this is the correct or the most natural way of looking at mixing processes, even in a statistical mechanical context. Entropy is, also in the statistical mechanical context, most naturally viewed as a function of a handful of macroscopic variables. Microscopic details only enter in the exact nature of those functional relationships.
